# Mr. Tomlinson's Case of Femoral Aneurism

**Published:** 1807-12

**Authors:** Thos. Tomlinson

**Affiliations:** Birmingham


### *To the Editors of the Medical and Physical Journal.*

GENTLEMEN,

PLEASE to insert in your Journal, the following case of successful operation for the cure of Femoral Aneurism, by tying the iliac artery, as recommended by Mr. Abernethy, with the exception of making use of only one ligature round the vessel.

### CASE.

Mr. Archer is about forty years of age and general good health; did not discover any unusual appearance of indisposition, until about four months ago; from which time he has observed the aneurismal tumour increase considerably; and, and, at the time of the operation, which was on the 29th of August, it was about the size of a large full grown apple.

The patient experienced so little inconvenience from the operations, that I did not make use of the lancet more than once, and then more from caution than necessity. On the 8th of September, the sutures were detached; on the 21st, the ligature came away; and, on the 24th, the patient was dismissed cured, and can walk a mile with the assistance of a stick. The tumour is decreased more than one half; and, from the first moment after the operation, the circulation of the blood was completely adequate to the purposes of the limb, which continued its natural warmth and sensation.

This instance of successful practice, added to those which have already been mentioned, must, with every dispassionate mind, recommend its adoption.

Here then, too, is a proof of one of the most deplorable maladies of the human constitution, and one of those which formerly admitted of no relief, submitting to the controul of our art; and, in this instance, an individual has been snatched from inevitable destruction, an affectionate father restored to his family, and a worthy member to society.

I do not mean to arrogate any undue praise to myself in relating the above case, but to shew to the world how rapidly our profession is advancing, and that the capability of performing the most complex and difficult operations is not confined to the metropolis.

I am, &c.

THOS. TOMLINSON.

*Birmingham, Oct.* 24, 1807.

